# Exploring the Italian Population’s attitudes toward health data sharing for healthcare purpose and scientific research: a cross-sectional study

**DOI:** 10.1093/pubmed/fdae313

**Published:** 2024-12-27

**Authors:** G Scaioli, G Lo Moro, M Martella, A Mara, M G Varì, C Previti, E Rolfini, A Scacchi, F Bert, R Siliquini

**Affiliations:** Department of Public Health and Pediatric Sciences, University of Turin, 10126 Turin, Italy; Department of Public Health and Pediatric Sciences, University of Turin, 10126 Turin, Italy; Department of Public Health and Pediatric Sciences, University of Turin, 10126 Turin, Italy; Department of Public Health and Pediatric Sciences, University of Turin, 10126 Turin, Italy; Department of Public Health and Pediatric Sciences, University of Turin, 10126 Turin, Italy; Department of Public Health and Pediatric Sciences, University of Turin, 10126 Turin, Italy; Department of Public Health and Pediatric Sciences, University of Turin, 10126 Turin, Italy; Department of Public Health and Pediatric Sciences, University of Turin, 10126 Turin, Italy; Department of Public Health and Pediatric Sciences, University of Turin, 10126 Turin, Italy; Department of Public Health and Pediatric Sciences, University of Turin, 10126 Turin, Italy; Molinette Hospital, AOU City of Health and Science of Turin, 10126 Turin, Italy

**Keywords:** attitudes, government and Law, health Data, health services, public health

## Abstract

**Background:**

This study aimed to explore the Italian population’s knowledge and perceptions regarding health data storage and sharing for treatment and research and to identify factors associated with citizens’ attitudes toward data storage and sharing.

**Methods:**

A cross-sectional questionnaire, distributed to 1389 participants, collected sociodemographic information, assessed knowledge and gauged attitudes toward sharing data for treatment and research. Descriptive analyses and logistic regressions were performed to examine the associations between sociodemographic factors and knowledge/attitudes about data storage and sharing.

**Results:**

Most respondents wrongly believed that healthcare providers could access personal health–related data across the entire national territory, while 94% expressed willingness to share personal health data nationwide. A substantial percentage of respondents (73%) fully agreed that storing and sharing personal health–related data could improve research and quality of care.

Males and younger individuals (<41 years) were likelier to have higher data-sharing knowledge. Lower educational-level respondents exhibited lower positive attitudes towards sharing health data for treatment and research purposes.

**Conclusions:**

The results provide valuable insights for policymakers, healthcare professionals and researchers seeking to improve data management, promote collaboration and leverage the full potential of health data for personalized care and scientific advancements.

## Introduction

The rapid digitization of healthcare systems has revolutionized how medical data are collected, stored and analyzed.^1^ Electronic health records, wearable devices and health monitoring applications have greatly expanded health data availability and accessibility. This wealth of information provides valuable insights for personalized healthcare and developing evidence-based medical interventions.^2^^,^^3^ The volume of available patient data grows exponentially each year.^4^ Still, this quantitative increase does not go hand in hand with improvements in data management quality, data sharing and integration and coordination among healthcare providers and institutions.^1^^,^^5^^,^^6^

The sharing of these health data holds significant importance for several reasons.^1^^,^^5^ Firstly, it plays a crucial role in addressing the healthcare needs of an aging population.^7^ As life expectancy continues to rise, it is essential to have a comprehensive understanding of individuals’ health status, medical history and treatment outcomes.^8^ By sharing health data, healthcare providers can collaborate more effectively and ensure continuity of care, especially for chronic patients, improving overall health outcomes and quality of life.^9^^,^^10^

Furthermore, the increasing specialization and fragmentation of medical practices necessitate seamless communication and integration of information across different healthcare professionals.^11^^,^^12^ Accessing and sharing comprehensive health data enables a holistic approach to patient care, facilitating better coordination among healthcare providers and reducing medical errors resulting from incomplete or fragmented information.^11^^,^^13–16^

Sharing health data also significantly enhances scientific research endeavors.^17^ By pooling diverse and comprehensive datasets, researchers can gain valuable insights into disease patterns, treatment outcomes and population health trends. This collaborative data-sharing approach fosters a collective knowledge base that benefits the scientific community. Sharing anonymized health data promotes transparency and reproducibility, allowing other researchers to validate and build upon existing findings.^18^^,^^19^

Data sharing also enables the exploration of rare diseases or conditions with limited individual datasets. By aggregating data from multiple sources, researchers can uncover patterns and gain a deeper understanding of these rare conditions, potentially leading to breakthroughs in diagnosis, treatment and prevention.^15^ Additionally, sharing health data promotes interdisciplinary collaboration between researchers from different fields, fostering a more holistic understanding and opening avenues for innovative research approaches.^1^^,^^5^

However, health data sharing is accompanied by concerns related to privacy and data security.^20^ The sensitive nature of health information raises legitimate concerns about unauthorized access, data breaches and potential misuse. Safeguarding patient privacy and maintaining confidentiality is paramount to ensuring public trust and confidence in the healthcare system.^21–24^ Another critical factor influencing health data sharing is citizens’ trust.^21^ To be willing to share their health information, individuals must have confidence that their data will be handled securely and used for legitimate purposes. Building and maintaining trust requires transparent practices, clear communication and robust privacy safeguards. Addressing the privacy concerns of individuals and establishing data protection measures are vital to foster trust and encourage data sharing for healthcare and scientific research.^25^

Several studies were conducted to investigate the citizens’ opinions and attitudes about health data sharing, most conducted in the USA before the pandemic.^25–32^ The studies demonstrated general support for using deidentified routinely collected data for health research and a willingness to share data to improve the quality of care. In contrast with these promising results, the abovementioned concerns lead to an inadequate use of healthcare data for treatment and research in various countries, including Italy.^9^^,^^10^ Despite the availability of vast amounts of health data, these significant barriers prevent their effective utilization. The potential benefits of digitizing healthcare systems, such as personalized healthcare and evidence-based interventions, still need to be fully realized due to challenges in data management, sharing and integration among healthcare providers and institutions.^9^^,^^20^^,^^22^^,^^24^ This lack of utilization hampers efforts to address the population’s healthcare needs, collaborate effectively among healthcare professionals and conduct robust scientific research. There is a pressing need to overcome these barriers and unlock the full potential of health data to improve healthcare delivery and scientific advancements.^9^^,^^24^

Therefore, this study aims to investigate the knowledge and perception of the general Italian population concerning the use of health data for care and scientific research purposes and to analyze factors that might be related to citizens’ attitudes on this topic. The aim is to assess whether the general population is willing to share personal and healthcare-related data for care and scientific purposes and to identify population factors that might predict a lower willingness to share these data. Specific aims of the study were to determine the percentage of individuals who are aware of the limited accessibility of personal healthcare data by healthcare providers, to assess whether citizens are willing to share personal health data within the whole national territory, to determine whether citizens believe that the storage and share of personal health data might be helpful for treatment and scientific purposes and to identify socio-demographic factors, such as age, gender, educational level and employment, associated with the abovementioned statements.

## Methods

A 20-item online questionnaire was designed for this cross-sectional study. The ethics committee of the University of Turin, Italy, approved the study. The study followed the guidelines for observational studies in epidemiology.^33^ The authors produced the questionnaire based on previous questionnaires.^25–32^^,^^34^ The questionnaire comprises a first section focusing on socio-demographic information, health-related information and information about internet use and a second section investigating knowledge and perceptions of the general population regarding sharing health data. In detail, the first section examined the age and gender of the participants, nationality, living conditions, educational level, employment and email and text messages to receive or send medical reports to the doctors. The second section explored the citizens’ knowledge about data sharing within the Italian context and their attitudes toward data sharing for treatment and scientific purposes. A pilot study, performed to assess the performance of the questionnaire, revealed no necessary changes; thus, pilot study results were included in the final analysis. The questionnaire was set on the Limesurvey (https://www.limesurvey.org/) platform and then shared online through social media, websites, mailing list of citizens’ rights associations (CittadinanzAttiva) and in person by paper flyers with a QR code distributed in outpatient rooms. Minors of 18 years were not allowed to participate in the study. Participation was voluntary and non-remunerated.

### Statistical analysis

The collected data were analyzed with STATA/SE 17.0 (Stata Corp., College Station, TX, USA). A descriptive analysis was performed for each variable using percentages and absolute numbers for categorical variables and means and standard deviations (SD) for continuous variables. Outcome variables on knowledge about healthcare data sharing (knowledge statement, KS) and attitudes about healthcare data sharing for care purposes and scientific research (attitude statement 1, AS1 and attitude statement 2, AS2, attitude statement 3, AS3 and attitude statement 4, AS4) were assessed. Supplementary Material (S1) shows details of the outcome variables. See online supplementary material for a color version of this figure. The outcome variables and several independent variables were dichotomized or categorized to increase the interpretability of the results. The outcome variables were dichotomized as follows: for the KS, the answers were dichotomized as correct answers versus all the other incorrect answers; for the AS1, the responses were dichotomized as ‘within the whole Italian territory’ versus all the other answers; and for the other attitude outcomes (AS2, AS3, AS4), the responses were dichotomized as ‘fully agree’ and ‘partially agree’ versus ‘fully disagree’ and ‘partially disagree’. Independent variables dichotomized/categorized were age (<41, 41–65, >65 years), residency (north of Italy versus center or south of Italy), size of the city in which the respondent lives (cities with >50 000 habitants versus cities with <50 000 habitants), marital status (married/cohabitant versus widowed, single, divorced), number of persons with which the respondent live (alone versus at least one person) and employment (student and employed versus homely, unemployed and retired). Univariable and multivariable regression analyses were performed for each outcome variable to assess factors potentially associated with higher knowledge/attitudes about data sharing. Variables with *P* < 0.25 at the univariable analysis were included in multivariable logistic regressions (stepwise backward process). A *P* < 0.05 was considered statistically significant.

## Results

A total of 1389 individuals completed the questionnaire and were included in the analysis. The percentage of females was 61.80%, and the mean age was 47.4 years (SD ± 10.7). The majority of participants were Italians (98.12%). Most participants were from the North of Italy (95.47%). Around 18% of the sample declared to live in a city with >250 000 inhabitants, while 14.5% declared to live in a town with <5000 inhabitants. Most participants were married (53.4%) and had at least a high school education (85.7%). A total of 30.35% of the interviewees worked in the healthcare sector.

For the outcome variable KS, the majority (65.4%) of the participants stated that healthcare providers might access the personal health–related data of each citizen in the whole national territory and 17% only within the region of residency. Almost 94% of the sample declared the willingness to share personal health–related data within the national territory (AS1). A total of 73% of the participants fully agreed with the statement that storing and sharing personal electronic health data might improve health-related research (AS2), and 73% fully agreed that storing and sharing personal health–related data might improve the quality of care (AS3). A total of 75.9% of the respondents fully agreed to allow personal healthcare data to be used for medical research by public entities (AS4). A detailed descriptive analysis is provided in Table 1.

**Table 1:** Descriptive analysis of the sample (*N* = 1389)

*Variable*		*N*	*%*
Gender	Female	858	61.8%
	Male	531	38.2%
Age (mean ± standard deviation)		47.39	±10.70
Nationality	Italian	1359	98.12%
	Foreign	26	1.88%
Region of residence	North	1308	95.47%
	Other	62	4.53%
Size of the hometown	>50 000 inhabitants	489	35.33%
	<50 000 inhabitants	895	64.67%
With how many people do you live?	I live alone	164	11.89%
	I do not live alone	1215	88.11%
Marital status	Cohabitant/Married	938	67.63%
	Single/Divorced/Widowed	741	32.37%
Education	Degree	637	45.93%
	High school	552	39.8%
	Middle school/Primary school	198	14,27%
Employment	Student/Employed	1200	85.52%
	Homely/Retired/Unemployed	187	13.48%
Do you study or work in the healthcare field?	No	966	69.65%
	Yes	421	30.35%
In your family, does someone work in the healthcare field?	No	946	68.20%
	Yes	441	31.80%
Do you take medicines regularly?	No	853	61.50%
	Yes	534	38.50%
KS: By presenting the European health card, is it possible for healthcare professionals from hospitals and local health authorities to access citizens’ health data?	Within the whole Italian territory	740	56.40%
	Within the region of residency of the citizen	211	16.08%
	Within the local health authority of residency of the citizen	146	11.13%
	Within the region of residency of the citizen in some regions, and within the local health authority of residence of the citizen in the other regions	103	7.85%
	Healthcare professionals cannot access citizens’ health data	112	8.54%
AS1: Would you like access to your health data by hospital and local health authority (ASL) operators to be possible by presenting your European health card:	within the whole Italian territory	1231	93.90%
	Within my region of residency	35	2.67%
	Within the local health authority of residency	23	1.75%
	I would like access to my health data by healthcare professionals to never be possible.	22	1.68%
AS2: Do you believe that sharing healthcare data in digital format (electronic, not paper-based) between healthcare entities (local health authorities, hospitals) and qualified research institutions can improve the quality of care? Please indicate your level of agreement:	Fully/partially agree Fully/partially disagree	1285 27	97.94% 2.06%
AS3: Do you believe storing and sharing healthcare data in digital format (electronic, not paper-based) between healthcare entities (local health authorities, hospitals) and qualified research institutions can improve medical/healthcare research? Please indicate your level of agreement:	Fully/partially agree Fully/partially disagree	1290 22	98.32% 1.68%
AS4: Would you agree to allow your healthcare data, processed in a completely anonymous form, to be used for medical research by public entities such as universities and research centers to provide information for better management of patients with the same condition/pathology as yours while ensuring total respect for privacy?	Fully/partially agree Fully/partially disagree	1273 40	96.95% 3.05%
If you answered ‘somewhat disagree’ or ‘completely disagree’ to the previous question, do you believe that this distrust applies only to your healthcare data or also to other types of data (e.g. economic data)?	I am against the sharing of any data. Yes, only for healthcare data	24 15	61.54% 38.46%
Have you ever used social networks (Facebook, Twitter), WhatsApp or SMS to send or receive medical reports?	No	518	39.45%
	Yes	762	58.04%
	I do not remember	33	2.51%
Have you ever used email to send or receive medical reports?	No	111	8.45%
	Yes	1181	89.95%
	I do not remember	21	1.60%

### Univariable and multivariable logistic regression models

**Fig. 1:**
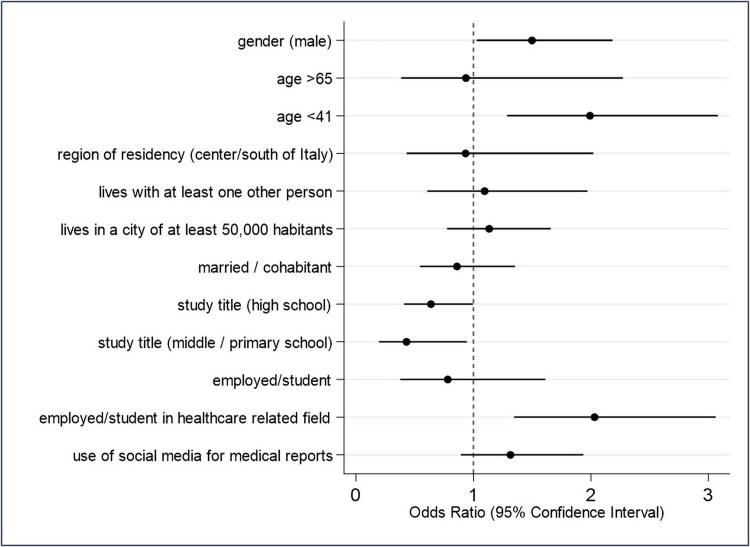
Multivariable logistic regression analysis. Outcome: knowledge about data sharing (KS): ‘by presenting the European health card, is it possible for healthcare professionals from hospitals and local health authorities to access citizens’ health data?’ (likelihood of correct answer versus wrong answers). The analysis included all the variables presented in the figure.

The model performed to assess factors potentially related to the knowledge of the population about sharing personal and health-related data (KS) showed that, after including independent variables, males and those in the category of age < 41 years had a higher likelihood of higher knowledge ((adjusted odds ratio [adjOR] 1.50, 95% confidence interval (CI) 1.03–2.18), and adjOR 1.99, 95% CI 1.29–3.08, respectively). Those with an educational level lower than a university degree had a lower likelihood of higher knowledge (high school adjOR 0.64, 95% CI 0.41–0.99; middle school or primary school adjOR 0.43, 95% CI 0.19–0.94). (Fig. 1 and Table Supplementary Material S2). See online supplementary material for a color version of this figure.

**Fig. 2:**
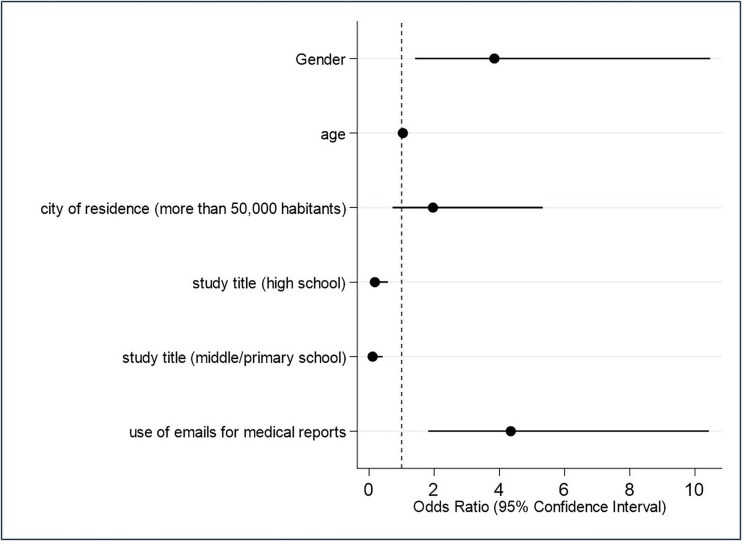
Multivariable logistic regression analysis. Outcome: Attitudes about data sharing 2 (AS2): ‘Do you believe that sharing healthcare data in digital format (electronic, not paper-based) between healthcare entities (local health authorities, hospitals) and qualified research institutions can improve the quality of care? Please indicate your level of agreement’ (likelihood of agreeing versus not agreeing with the statement). The analysis included all the variables presented in the figure.

**Fig. 3:**
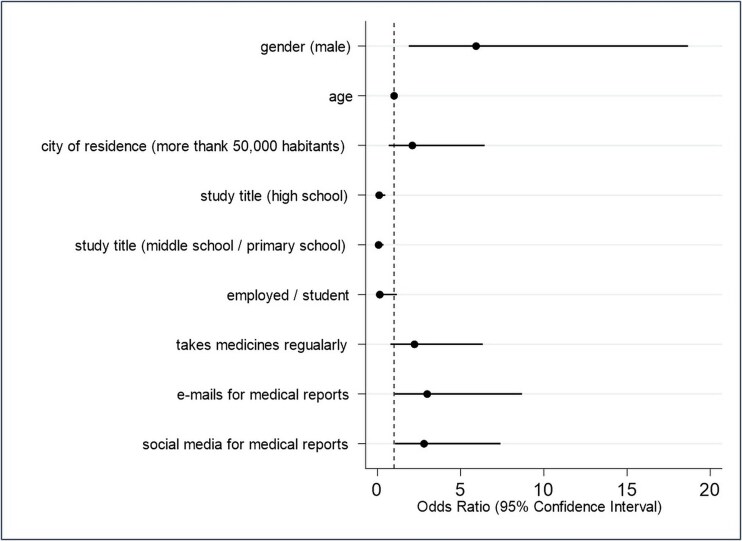
Logistic regression analysis. Outcome: Attitudes about data sharing 3 (AS3): ‘Do you believe that storing and sharing healthcare data in digital format (electronic, not paper-based) between healthcare entities (local health authorities, hospitals) and qualified research institutions can improve medical/healthcare research? Please indicate your level of agreement’ (likelihood of agreeing versus not agreeing with the statement). The analysis included all the variables presented in the figure.

Concerning the attitude outcomes, after adjusting for independent variables, the age category < 41 years was associated with a higher likelihood of the willingness to share health data within the whole national territory than the other answers (outcome variable AS1, adjOR 1.55, 95% CI 1.21–5.35) (Table Supplementary Material S3). See online supplementary material for a color version of this figure. About the outcome AS2, those with an educational level lower than a university degree had a lower likelihood of positive attitudes about this outcome (adjOR 0.18, 95% CI 0.05–0.58 for high school and adjOR 0.11, 95% CI 0.03–0.41 for middle school or lower). Those who declared to use email to receive or send medical reports to doctors had a higher likelihood of positive attitudes about this outcome (adjOR 4.34, 95% CI 1.81–10.42) (Fig. 2 and Table Supplementary Material S4). See online supplementary material for a color version of this figure. Concerning the outcome AS3, those with an educational level lower than a university degree had a lower likelihood of positive attitudes about this outcome (adjOR 0.10, 95% CI 0.02–0.48 for high school and adjOR 0.07, 95% CI 0.01–0.37 for middle school or lower). Those who declared to use email or social networks to contact healthcare professionals have a higher likelihood of positive attitudes about this outcome (adjOR 2.99, 95% CI 1.03–8.69 and adjOR 2.80, 95% CI 1.06–7.40, respectively) (Fig. 3 and Table Supplementary Material S5). See online supplementary material for a color version of this figure. Considering the outcome variable AS4, the multivariable model showed that those with a low educational level had a lower likelihood of agreement with the AS4 statement when compared with those with a university degree (OR 0.39, 95% CI 0.17–0.93 for high school and OR 0.17, 95% CI 0.06–0.43 for middle school/primary school). Moreover, those who declared to use social media to send or receive medical reports had a higher likelihood of agreeing with the outcome (OR 1.97, 95% CI 1.02–3.80) (Table Supplementary Material S6). See online supplementary material for a color version of this figure.

## Discussion

### Main findings of this study

This study aimed to investigate the knowledge and perception of the general Italian population regarding the use of health data for treatment and scientific research purposes and to identify factors associated with citizens’ attitudes toward data sharing. The findings provide insights into the general population’s willingness to share personal and health data for treatment and scientific purposes and identify characteristics that may predict a lower willingness to store and share such data.

Most respondents believed that access to health-related data of the citizens could be possible by healthcare providers for care purposes within the whole Italian territory. That is not true, given that Italy has a fragmented healthcare system, with the majority of the responsibilities being in charge of the Italian regions and the central government with a role of guidance and control^35^ This leads to different healthcare systems with different and, often, not interoperable information systems.^36^^,^^37^ Therefore, most healthcare data are accessible only within a specific region, but most of the time only within the local health authority of residence of the citizen, given that each local health authority has a different information system that is not interoperable with the systems of the other local health authorities, even within the same Region. The introduction of the electronic personal health record (EPHR),^38^ a tool that allows storing all the healthcare data of a citizen, might represent an improvement in terms of collecting and sharing personal health data; however, recent statistics demonstrated that the use of EPHR in Italy is still shallow.^39^^,^^40^

Despite this poor knowledge about sharing health-related data, the respondents demonstrated a high willingness to share personal health data and information within the Italian territory (93.9%). This willingness serves as an encouragement to increase the possibility of sharing health personal data within the entire Italian territory for treatment and research purposes, given that 76% of the sample declared to agree to allow their health data, processed in a completely anonymous form, to be used for healthcare research purposes by public entities such as universities and research centers. A total of 73% of the respondents fully agree with the statement that storing and sharing personal electronic health data might improve health-related studies, and 73% fully agree with the statement that storing and sharing personal health-related data might improve the quality of care.

The logistic regression analyses highlighted the influence of socio-demographic characteristics and educational background on knowledge and attitudes toward health data sharing. Males and younger individuals have a higher level of knowledge on this topic, indicating greater exposure to information and technology.^41^^,^^42^ Higher education levels and the use of technology to communicate with doctors are associated with higher knowledge levels, suggesting that education and professional background play a role in understanding health data sharing.

### What is already known on this topic

Other studies on this topic showed controversial results. A pan-European survey conducted in 2016 showed that the European population’s willingness to store electronic health data was generally preferred. Still, there are worries about broader access to and sharing of this information.^25^ A survey conducted in the USA showed that the majority (66%) of the respondents would be willing to participate in biobank research for open-ended research use and widespread sharing of their biosamples and data.^27^ Another US study demonstrated that, for US citizens, the essential benefits of sharing data were ‘helping my doctor make better decisions about my health’ and ‘helping make new therapies available faster’. In contrast, the most crucial data-sharing risk identified was health data being ‘stolen by hackers’. Moreover, for the respondents who were not comfortable with researchers accessing their de-identified data, most reported that their comfort levels would increase if they were able to learn how their data were protected.^28^

Regarding the multivariable logistic regression results, several other studies found that educational level is associated with knowledge and attitudes about health topics.^43–45^

### What this study adds

The present study sheds light on the influence of educational level and digital communication with healthcare professionals on attitudes toward data sharing. Its findings might have broader implications for an international audience despite focusing on a specific Italian sample. Increased (health and digital) literacy and capacity building have been identified worldwide as fundamental elements for promoting change within populations and enabling informed health decisions.^46^ In this context, enhancing the population’s education level is crucial to improving individuals’ and communities’ ability to understand and use health information effectively.^47^ Targeted interventions that enhance understanding of the benefits of health data sharing can help accept more collaborative health practices, overcoming resistance linked to educational disparities. These findings are beneficial for developing capacity-building policies that encourage change at the international level.^48^

In addition to capacity building, enabling factors, e.g. characteristics of the environment that facilitate action and the skill or resource required to attain specific behavior, are essential for raising attitude and behavior changes within populations.^49^ In the context of health data sharing, establishing transparent frameworks and trustworthy systems becomes a fundamental responsibility for policymakers. Policymakers can promote health data sharing by shaping environments that promote openness and safeguard data privacy, helping individuals feel confident about using and protecting their personal information.^50^

Despite the abovementioned differences, the high percentage of individuals willing to share their health data represents a positive factor.^31^ Such openness among the population suggests a readiness to support collaborative health initiatives, which can significantly enhance healthcare quality, continuity of care and research advancements. This willingness provides a foundation for policies that promote secure and transparent health data-sharing frameworks, ensuring that personal information is accessible only to qualified institutions while fully respecting data protection regulations.^18^^,^^34^

### Limitations of the study

The study presents some limitations. The collected data were self-reported, which may introduce bias. Additionally, it should be noted that this sample does not fully represent the Italian population. Specifically, individuals from Northern Italy with at least a high school diploma were overrepresented, while foreign residents were underrepresented.^45^ Furthermore, the online distribution does not allow persons without an internet connection to answer the questionnaire. However, recent statistics demonstrated that the overwhelming majority of the Italian population has a smartphone with an internet connection^45^; Moreover, the ‘promotion’ of the questionnaire in the outpatient waiting rooms allows a broader audience to acknowledge the study and the inclusion of a more representative sample size.

## Conclusions

In conclusion, the present study shows that the population mainly favors sharing health data. Data sharing in healthcare is crucial for several public interest factors and treatment goals. The scarcity of pertinent and reliable scientific information to support ‘evidence-based’ judgments is one of the critical challenges in decision-making processes in both the clinical and health policy fields. Regulatory obstacles must be cleared, and data security and quality must be guaranteed for data sharing to realize its full potential. With its enormous potential, it is time to update healthcare data management regulations so that a vast amount of helpful information can be processed and studied for research purposes. The timing seems right to formalize an alliance and complementarity between those engaged in research and those involved in data protection.

## Supplementary Material

supplementary_material_revised_clean_pubmed_fdae313

## Data Availability

The article’s data will be shared at a reasonable request by the corresponding author.

## Notes

**G. Scaioli,** Associate Professor, MD

**G. Lo Moro,** Assistant Professor, MD

**M. Martella,** MD

**A. Mara,** MD

**M.G. Varì,** MD

**C. Previti,** MD

**E. Rolfini,** Medical residents, MD

**A. Scacchi,** MD

**F. Bert,** Full Professor, MD

**R. Siliquini,** Full Professor, MD
