# Supplementary material for: Exploring the Italian Population’s attitudes toward health data sharing for healthcare purpose and scientific research: a cross-sectional study
Source: J Public Health (Oxf). 2024 Dec 27;47(1):99–108. doi: 10.1093/pubmed/fdae313 (PMC11982610; doi:10.1093/pubmed/fdae313)
Supplement: supplementary_material_revised_clean_pubmed_fdae313 [file supplementary_material_revised_clean_pubmed_fdae313.docx]

**SUPPLEMENTARY MATERIAL**

**S1. Description of the outcome variables:**

The questions/statements included as outcomes were “By presenting the European health card, is it possible for healthcare professionals from hospitals and local health authorities to access citizens' health data” (knowledge statement, KS), “Would you like access to your health data by hospital and local health authority (ASL) operators to be possible by presenting your European health card” (attitude statement 1, AS1), “Do you believe that sharing healthcare data in digital format (electronic, not paper-based) between healthcare entities (local health authorities, hospitals) and qualified research institutions can improve the quality of care? Please indicate your level of agreement” (attitude statement 2, AS2), “Do you believe that storing and sharing healthcare data in digital format (electronic, not paper-based) between healthcare entities (local health authorities, hospitals) and qualified research institutions can improve medical/healthcare research? Please indicate your level of agreement” (attitude statement 3, AS3), “Would you agree to allow your healthcare data, processed in a completely anonymous form, to be used for medical research by public entities such as universities and research centers, to provide information for better management of patients with the same condition/pathology as yours, while ensuring total respect for privacy?” (attitude statement 4, AS4). The answer for the KS was “Within the Region of residency of the citizen in some Regions, and within the Local Health Authority of residency of the citizen in the other Regions” (correct answer), “within the whole Italian territory,” “Within the Region of residency of the citizen,” “Within the local health authority of residency of the citizen,” “Healthcare professionals cannot access citizens' health data” (wrong answers).

The answers for the first attitude statement (AS1) were “within the whole Italian territory,” “within the whole Italian territory,” “Within the local health authority of residency,” and “I would like access to my health data by healthcare professionals to never be possible.” For the other attitude statements (AS2, AS3, AS4), the answers were “fully agree,” “partially agree,” “partially disagree,” and “fully disagree.”

**Table S2. Univariable e multivariable logistic regression analysis. Outcome: knowledge about data sharing (KS)**

| **by presenting the European health card, can healthcare professionals from hospitals and local health authorities access citizens' health data? (correct answer vs wrong answers)** | | **Univariable** | | **Multivariable*** | |
| --- | --- | --- | --- | --- | --- |
|  |  | **Odds Ratio** | **Confidence Interval 95%** | **Odds Ratio** | **Confidence Interval 95%** |
| Gender | Female | ref | | ref | |
|  | Male | 1.18 | 0.83 – 1.67 | **1.50** | **1.03 – 2.18** |
| age | 41-65 | ref | | ref | |
|  | >65 | 1.28 | 0.62 - 2.65 | 0.93 | 0.38 – 2.27 |
|  | <41 | **3.31** | **2.30 – 4.76** | **1.99** | **1.29 – 3.08** |
| Nationality | Foringer | ref | | / | |
|  | Italian | 0.79 | 0.18 - 3.44 | / | |
| Region of residency | North of Italy | ref | ref | ref | ref |
|  | Other | 0.58 | 0.28-1.22 | 0.93 | 0.43-2.02 |
| Number of people you live with | alone | ref | ref | ref | ref |
|  | With at least one other person | **1.71** | **1.07-2.73** | 1.09 | 0.61-1.97 |
| Size of the place in which you live | More than 50,000 inhabitants | **1.75** | **1.24-2.47** | 1.13 | 0.77-1.66 |
|  | Less than 50,000 inhabitants | ref | | ref | |
| Marital status | Married / cohabitant | **0.56** | **0.40-0.80** | 0.86 | 0.54-1.35 |
|  | Widowed/ single | ref | | ref | |
| Study title | University degree | ref | | ref | |
|  | High school | **0.40** | **0.28-0.60** | **0.64** | **0.41-0.99** |
|  | Middle school/primary school | **0.24** | **0.11-0.51** | **0.43** | **0.19-0.94** |
| Employment | Employed / student | 1.49 | 0.84-2.65 | 0.78 | 0.38-1.61 |
|  | Unemployed/ homely/retired | ref | | ref | |
| Study or work in the healthcare-related field | no | ref | | ref | |
|  | yes | **2.96** | **2.09 – 4.20** | **2.03** | **1.34-3.06** |
| Do you take medicines regularly | no | ref | ref | / | / |
|  | yes | 0.98 | 0.69-1.39 | / | / |
| Have you ever used email to send or receive medical reports? | No /do not remember | ref | | / | / |
|  | yes | 1.16 | 0.64-2.12 | / | / |
| Have you ever used social media (Facebook, Twitter), WhatsApp, or SMS to send or receive medical reports? | No /do not remember | ref | | ref | |
|  | yes | 1.49 | 1.04-2.15 | 1.31 | 0.89-1.93 |

* adjusted for all the factors included in the multivariable results

**Table S3. Logistic regression analysis. Outcome: Attitudes about data sharing 1 (AS1)**

| **“Would you like access to your health data by hospital and local health authority (ASL) operators to be possible by presenting your European health card”** | | **Univariable** | | **Multivariable*** | |
| --- | --- | --- | --- | --- | --- |
|  |  | Odds Ratio | Confidence Interval 95% | Odds Ratio | Confidence Interval 95% |
| Gender | Female | ref | | ref | |
|  | Male | 1.63 | 0.99-2.68 | 1.63 | 0.99-2.69 |
| Age category | 41-65 | ref | | ref | |
|  | >65 | 1.81 | 0.64-5.73 | 1.82 | 0.64-5.13 |
|  | <41 | 2.74 | 1.35-5.56 | **1.55** | **1.21-5.35** |
| Region of residency | North of Italy | 0.91 | 0.28-2.99 | / | / |
|  | other | ref | | / | / |
| Number of people you live with | alone | 0.90 | 0.45-1.80 | / | / |
|  | With at least one other person | ref | | / | / |
| Size of the place in which you live | More than 50,000 inhabitants | 1.08 | 0.67-1.74 | / | / |
|  | Less than 50,000 inhabitants | ref | | / | / |
| Marital status | Married / cohabitant | 0.96 | 0.59-1.57 | / | / |
|  | Widowed/ single | ref | | / | / |
| Study title | University degree | ref | | ref | |
|  | High school | 0.87 | 0.52-1.44 | 1.05 | 0.62-1.79 |
|  | Middle school/elementary school | 0.52 | 0.28-0.96 | 0.66 | 0.35-1.25 |
| Employment | Employed / student | 0.82 | 0.40-1.68 | / | / |
|  | Unemployed/ homely/retired | ref | | / | / |
| Study or work in the healthcare-related field | no | ref | | / | / |
|  | yes | 0.95 | 0.58-1.55 | / | / |
| Do you take drugs regularly? | no | ref | | / | / |
|  | yes | 1.06 | 0.66-1.69 | / | / |
| Have you ever used email to send or receive medical reports? | No /do not remember | ref | | / | / |
|  | yes | 0.85 | 0.38-1.88 | / | / |
| Have you ever used social media (Facebook, Twitter), WhatsApp, or SMS to send or receive medical reports? | No /do not remember | ref | | / | / |
|  | yes | 1.27 | 0.80-1.99 | / | / |
| Do you take drugs regularly? | no | ref | | / | / |
|  | yes | 1.14 | 0.53-2.41 | / | / |

* adjusted for all the factors included in the multivariable results

**Table S4. Logistic regression analysis. Outcome: Attitudes about data sharing 2 (AS2)**

| **“Do you believe that sharing healthcare data in digital format (electronic, not paper-based) between healthcare entities (local health authorities, hospitals) and qualified research institutions can improve the quality of care? Please indicate your level of agreement.” (agree vs. do not agree)** | | **Univariabile** | | **Multivariabile*** | |
| --- | --- | --- | --- | --- | --- |
|  |  | **Odds Ratio** | **Confidence Interval 95%** | **Odds Ratio** | **Confidence Interval 95%** |
| Gender | Female | ref | | ref | |
|  | Male | **2.85** | **1.07-7.60** | **3.84** | **1.41-10.46** |
| age |  | 1.00 | 0.97-1.04 | 1.03 | 0.99-1.08 |
| With how many people do you live | alone | 1.58 | 0.37-6.77 | / | / |
|  | With at least one other person | ref | | ref | |
| Size of the place in which you live | More than 50,000 inhabitants | 2.44 | 0.92-6.50 | 1.96 | 0.72-5.33 |
|  | Less than 50,000 inhabitants | ref | | ref | |
| Marital status | Married / cohabitant | 0.74 | 0.31-1.76 | / | / |
|  | Widowed/ single | ref | | ref | |
| Study title | University degree | ref | | ref | |
|  | High school | **0.22** | **0.07-0.67** | **0.18** | **0.05-0.58** |
|  | Middle school/elementary school | **0.14** | **0.04-0.47** | **0.11** | **0.03-0.41** |
| Employment | Employed / student | 0.82 | 0.24-2.75 | / | / |
|  | Unemployed/ homely/retired | ref | | ref | |
| Study or work in the healthcare-related field | no | ref | | ref | |
|  | yes | 1.24 | 0.52-2.96 | / | / |
| Do you take drugs regularly? | no | ref |  | ref | |
|  | yes | 0.68 | 0.31-1.45 | / | / |
| Have you ever used email to send or receive medical reports? | No /do not remember | ref | | ref | |
|  | yes | **3.94** | **1.69-9.19** | **4.34** | **1.81-10.42** |
| Have you ever used social media (Facebook, Twitter), WhatsApp, or SMS to send or receive medical reports? | No /do not remember | ref | | ref | |
|  | yes | 1.50 | 0.67-3.21 | / | / |

* adjusted for all the factors included in the multivariable results

**Table S5. Logistic regression analysis. Outcome: Attitudes about data sharing 3 (AS3)**

| **“Do you believe that storing and sharing healthcare data in digital format (electronic, not paper-based) between healthcare entities (local health authorities, hospitals) and qualified research institutions can improve medical/healthcare research? Please indicate your level of agreement.”** | | **Univariable** | | **Multivariable*** | |
| --- | --- | --- | --- | --- | --- |
|  |  | **Odds Ratio** | **Confidence Interval 95%** | **Odds Ratio** | **Confidence Interval 95%** |
| Gender | Female | ref | | ref | |
|  | Male | 2.91 | 0.98-8.66 | 5.92 | 1.88-18.66 |
| age | | 0.99 | 0.96-1.03 | 1.00 | 0.94-1.07 |
| Region of residency | North of Italy | 1.12 | 0.15-8.51 | / | / |
|  | other | ref |  | / | / |
| Number of people you live with | alone | 2.65 | 0.35-19.87 | / | / |
|  | With at least one other person | ref |  | ref |  |
| Size of the place in which you live | More than 50,000 inhabitants | 2.49 | 0.84-7.41 | 2.09 | 0.68-6.44 |
|  | Less than 50,000 inhabitants | ref |  | ref |  |
| Marital status | Married / cohabitant | 0.79 | 0.30-2.04 | / | / |
|  | Widowed/ single | ref |  | ref |  |
| Study title | University degree | ref |  | ref |  |
|  | High school | **0.11** | **0.03-0.52** | **0.10** | **0.02-0.48** |
|  | Middle school/elementary school | **0.09** | **0.02-0.47** | **0.07** | **0.01-0.37** |
| Employment | Employed / student | 0.31 | 0.04-2.31 | 0.14 | 0.02-1.17 |
|  | Unemployed/ homely/retired | ref |  | ref |  |
| Study or work in the healthcare-related field | no | ref |  | / | / |
|  | yes | 1.96 | 0.66-5.84 | / | / |
| Do you take drugs regularly? | no | ref |  | ref |  |
|  | yes | 2.18 | 0.80-5.95 | 2.22 | 0.78-6.33 |
| Have you ever used email to send or receive medical reports? | No /do not remember | ref |  | ref |  |
|  | yes | **3.46** | **1.33-9.01** | **2.99** | **1.03-8.69** |
| Have you ever used social media (Facebook, Twitter), WhatsApp, or SMS to send or receive medical reports? | No /do not remember | ref |  | ref |  |
|  | yes | **3.01** | **1.22-7.44** | **2.80** | **1.06-7.40** |

* adjusted for all the factors included in the multivariable results

**Table S6. Logistic regression analysis. Outcome: Attitudes about data sharing 4 (AS4)**

| **“Would you agree to allow your healthcare data, processed in a completely anonymous form, to be used for medical research by public entities such as universities and research centers to provide information for better management of patients with the same condition/pathology as yours, while ensuring total respect for privacy?”** | | **Univariable** | | **Multivariable*** | |
| --- | --- | --- | --- | --- | --- |
|  |  | **Odds Ratio** | **Confidence Interval 95%** | **Odds Ratio** | **Confidence Interval 95%** |
| Gender | Female | ref | | ref | |
|  | Male | 1.50 | 0.76-2.98 | 1.90 | 0.94-3.85 |
| age |  | 1.00 | 0.97-1.03 | 1.03 | 0.99-1.06 |
| Nationality | Foringer | ref | | ref | |
|  | Italian | 0.30 | 0.68-1.34 | 0.35 | 0.07-1.66 |
| Number of people you live with | alone | 1.15 | 0.41-3.28 | / | / |
|  | With at least one other person | ref | | / | / |
| Size of the place in which you live | More than 50,000 inhabitants | 1.14 | 0.58-2.23 | / | / |
|  | Less than 50,000 inhabitants | ref | | / | / |
| Marital status | Married / cohabitant | 0.61 | 0.29-1.29 | 1.05 | 0.71-1.55 |
|  | Widowed/ single | ref | | ref | |
| Study title | University degree | ref | | ref | |
|  | High school | 0.47 | 1.18-0.93 | **0.39** | **0.17-0.93** |
|  | Middle school/elementary school | 0.19 | 0.08-0.45 | **0.17** | **0.06-0.43** |
| Employment | Employed / student | 0.53 | 0.16-1.72 | / | / |
|  | Unemployed/ homely/retired | ref | | / | / |
| Study or work in the healthcare-related field | no | ref | | ref | |
|  | yes | 2.07 | 0.91-4.73 | 1.54 | 0.64-3.73 |
| Do you take drugs regularly? | no | ref | | / | / |
|  | yes | 1.33 | 0.68-2.60 | / | / |
| Have you ever used email to send or receive medical reports? | No /do not remember | ref | | / | / |
|  | yes | 1.61 | 0.66-3.90 | / | / |
| Have you ever used social media (Facebook, Twitter), WhatsApp, or SMS to send or receive medical reports? | No /do not remember | ref | | ref | |
|  | yes | **1.91** | **1.01-3.61** | **1.97** | **1.02-3.80** |

* adjusted for all the factors included in the multivariable results
